# Visuospatial Function at Sub-Acute Phase Predicts Fatigue 10 Years After Stroke

**DOI:** 10.3389/fneur.2020.562706

**Published:** 2020-10-23

**Authors:** Eva Elgh, Xiaolei Hu

**Affiliations:** ^1^Department of Psychology, Umeå University, Umeå, Sweden; ^2^Department of Community Medicine and Rehabilitation, Umeå University, Umeå, Sweden

**Keywords:** fatigue, visuospatial function, block design, stroke, multivariate analysis, longitudinal study, predictor

## Abstract

**Background and Objective:** Fatigue is common among stroke survivors; and has significant negative consequences. However, long-term follow-up on post-stroke fatigue and it's association with cognitive and physiological parameters remains vague.

**Methods:** A prospective cohort study was carried out on 38 young stroke survivors (aged 18–65 at stroke onset) living in the community 10 years after first-ever stroke. Fatigue was assessed by Fatigue assessment scale (FAS). Global cognition and cognitive sub-domains were assessed repeatedly at 1 week, 7 months, and 10 years after their first-ever stroke. Univariate correlation analysis was used to investigate associations and multivariate regression was used to investigate predictors and association with fatigue.

**Results:** At 10-years follow-up after stroke onset, more than half of the 38 participants suffered from fatigue [with median score 25 on FAS with 25–75% percentile (21–28)]. Most of them were independent in their everyday life [mRS median score 1 (0–2)]. In univariate correlation analyses, higher fatigue score was significantly correlated to higher independence in the daily activity, higher BMI, anxiety, higher scores on global cognition and better working memory at 10-years follow-up as well as better visuospatial functions after 7 months and 10-years. In a multiple regression analysis, only visuospatial function at 7-months follow-up was a significant predictor of fatigue 10 years after stroke onset [*F* = 23.07, *p* < 0.009], with adjusted (*R*^2^ = 0.815) i.e., higher scores on Block design were associated with more fatigue.

**Conclusion:** Our results extended the time course of post-stroke fatigue up to 10 years after stroke onset. The participants with more fatigue performed better in cognitive assessments and daily activity, which indicated dissociation between fatigue and fatigability among stroke patients. Visuospatial function at the sub-acute phase predicted independently late post-stroke fatigue. This may offer a broad time window for rehabilitation and information about fatigue. The clinical implications of the current findings are worth to be studied further.

## Introduction

Fatigue is a common disabling symptom following stroke. It occurs not only in the early phase but also in the chronic phase after stroke (1, 2). Post-stroke fatigue is an independent predictor of shorter survival (3, 4), institutionalization (3, 5), poorer functional outcome (6), and greater dependency for daily activity (7). However, studies of post-stroke fatigue have often been carried out within 2–3 years after stroke onset. Very long-term follow-up on post-stroke fatigue has been rarely reported (8).

Fatigue is often examined subjectively with self-assessment questionnaires and objective measure of fatigue are challenging (9). Kluger et al. suggests a distinction between a person's own perceived fatigue (fatigue) and objective fatigue defined as a deterioration in performance when performing a mental or physical task (fatigability) (10). Fatigability could possibly be assessed with various objective motor and cognitive tests (9). However, knowledge of objective assessments of fatigue is largely lacking.

Furthermore, many clinical parameters, emotional and cognitive experiences have been suggested to play a role for fatigue in patients with stroke (2). Knowledge of factors underlying post-stroke fatigue may supply important information regarding treatment strategies. However, it's still difficult to predict the extent and duration of post-stroke fatigue.

The aim of the current study was to investigate the occurrence of fatigue 10 years after stroke onset, and assess potential relationship between fatigue and cognition as well as other clinical characteristics of participants among young stroke survivors in the unique longitudinal design.

## Materials and Methods

### Study Design

This study is a single-center prospective cohort study of stroke survivors with three consecutive follow-ups over a 10-years period after a first-ever stroke. It was carried out at the Department of Neurological Rehabilitation, University Hospital of Umeå, Sweden. Ethical approval was obtained from the regional Ethical Review Board in Umeå, Sweden, D-nr 2015/144-31.

### Recruitment and Participants

All young patients (>18 years to 65 years) who had suffered a first-ever stroke between January 2004 and December 2007 with neuropsychological assessments (NPAs) within the first year after stroke, at Stroke Centrum, University Hospital of Umeå, Sweden were contacted. They were provided informed and written consent via letter and telephone for recruitment to the study. Exclusion criteria were severe dementia, severe aphasia, and severe comorbidity/non-community-dwelling, recurrence of stroke/TIA and other physical and/or psychiatric disease after first-ever stroke. Out of 108 first-ever young stroke patients with acute NPAs, 38 stroke survivors with previous NPAs participated in the 10-years follow-up after a thorough recruitment process between 2015 and 2016 (11). The participants provided their written informed consent to participate in this study. All participants were native Swedish citizens.

### Data Collection

Baseline data were collected from the Riksstroke registry and medical records. Participants also provided information regarding their education level, social status, information about stroke-onset and employment. They completed various questionnaires at home 1 month prior to the scheduled appointment for NPAs.

### Questionnaires

#### Fatigue Assessment

Fatigue assessment scale (FAS) is a self-assessment questionnaire used for identifying symptoms of chronic fatigue (12). It consists of ten questions regarding both physical and mental problems regarding fatigue and has a maximum score of 50 points. Higher scores indicate higher degree of fatigue. Cutoff was set at ≥ 24 points and scores over that was an indication that the patient had post-stroke fatigue (13).

#### Depression and Anxiety Assessments

Depression and anxiety were assessed with the Beck Depression Inventory-II (BDI-II) (14) and Beck Anxiety Inventory (BAI) (15).

#### Daily Activity Assessment

The modified Rankin Scale questionnaire (mRSq) is a simple questionnaire for assessment of daily activity including motor function after stroke. Five questions are to be answered “yes” or “no” by the patient and then modified into a scale with five categories from 0 (no symptoms) to 5 (total physical dependence). mRS scores ≤2 are considered as total independence (16). The mRSq is validated for use after stroke (16, 17).

#### Cognitive Function Assessments

The neuropsychological assessments were completed by four assessors who were blinded to previous assessments. The entire test battery took ~2–3 h, with a 30-min break with refreshments in the middle. To ensure comparability, the selection of tests at follow-up was based on the tests that had been used at the first assessment within 1 year after stroke. Notably, Wechsler Adult Intelligence Scale (WAIS)-R and WAIS-III was replaced by WAIS-IV at 10-years follow-up due to practical reasons. Previous validation studies have shown that WAIS-IV has the same construction as WAIS-III/R (18), with very high correlation between subscales (r = 0.82–94) (19). In addition, Mini mental state examination (MMSE) was carried out immediately prior to NPAs.

The following cognitive domains were presented in the present study: process speed *(TMT-A)*, visuospatial function *(Block design from WAIS)*, executive function (*TMT-B*), working memory (*Digit Span from WAIS*) (Table 2). Alterations of cognitive domains over 10-year follow-up have been presented in detail previously (11).

### Data Presentation and Statistical Analysis

Demographic characteristics are presented as mean ± SD, number and number of cases (%) or median [25–75% interquartile ranges (IQR)] as appropriate. Baseline characteristics in patients were compared using a Mann-Whitney test, Fisher's exact test, or Chi-square test when appropriate.

Data from NPA are presented in raw median scores with 25–75% IQR because of the limited and varying number of participants at the early stage. The raw scores of the NPAs were presented at three time-points at acute stage (1 week after stroke), sub-acute stage (7-months after stroke) and chronic stage (10-years after stroke) in Table 2. No adjustment was made for missing values. Mann-Whitney test was chosen for non-parametric comparisons between groups with (FAS score > 24) or without (FAS < 24) fatigue. A non-parametric Spearman correlation with two-tailed test was used to obtain the analysis univariate correlations. Simple linear regression was used to predict fatigue based on a significant independent variable. Statistical analyses above were performed using GraphPad Prism software version 8.0.

The significant variables in univariate correlation analyses were included in multivariate linear regression analysis. In multivariate linear regression, a stepwise method was used to find the significant variables that may predict fatigue. Tolerance's value and VIF was presented to show whether collinearity existed. To determine the strength of the correlation for each separate variable, we used standardized coefficients. *R*^2^ was examined to indicate the amount of fatigue explained by the variables in the model. Multivariate linear regressions were performed by using IBM SPSS statistics. A *p* < 0.05 was considered significant.

## Results

### Basic Demographic and Clinical Characteristics

Baseline characteristics of study participants at stroke-onset, participant with and without fatigue at stroke onset are presented in Table 1. The mean age of participants was 53.9 (SD 9.1) years at stroke-onset. All participants were native Swedish citizens who living in the community. The dominate stroke sub-type was ischemia (79%). Left- and right hemisphere stroke were evenly distributed among the participants. The most common cerebrovascular risk factor was hypertension (29%).

**Table 1:** Demographic and clinical characteristics of all participants at stroke-onset, their correlations with fatigue, and comparation between groups with and without fatigue.

**Patient characteristics at stroke-onset**		**Univariate correlation with fatigue**		**Comparisons between groups** **with and without fatigue**		
	**Total**	**r**	***p*-value**	**FAS > 24**	**FAS < 24**	***p*-value**
Age (mean ± SD)	53.9 ± 9.1	−0.00	0.99	50.6 ± 11.5	52.6 ± 9.3	0.79
Gender (Men/Women)	19/19	0.04	0.87	6/5	4/4	>0.99
Risk factors [number of case (%)]						0.14
Hypertension	11 (29%)	0.18	0.46	3 (27%)	1 (13%)	
Diabetes	4 (11%)	0.37	0.12	3 (27%)	0	
Atrial fibrillation	4 (11%)	−0.33	0.17	0	1 (13%)	
Location of stroke (left-/right hemisphere)	19/19	−0.25	0.30	4/7	3/5	>0.99
Stroke sub-type [number of case (%)]		0.04	0.87			>0.99
Ischemia	30 (79%)			8 (73%)	6 (75%)	
Hemorrhage	6 (16%)			2 (18%)	2 (25%)	
Unknown	2 (5%)			1 (9%)	0 (0%)	

At 10-years follow-up (Table 2), most of the participants (79%) lived together with somebody. Almost half of the participants (*n*= 17, 17/37, 46%) had more than 12 years education. Approximately two third of participants were overweight (BMI > 25) with mean 23.6 (SD 9.5) for the whole group. More than one fourth of the participants worked still on part-time or full-time job at 10-year follow-up.

**Table 2:** Demographic and clinical characteristics of all participants at 10-years follow-up, their correlations with fatigue, and comparation between groups with and without fatigue.

**Patient characteristics at 10-years follow-up**		**Univariate correlation with fatigue**		**Comparisons between groups** **with and without fatigue**		
	**Total**	***r***	***p*-value**	**FAS > 24**	**FAS < 24**	***p*-value**
Age (mean ± SD)	63.8 ±10.6	−0.12	0.62	60.8 ± 12.8	65.1 ± 8.0	0.58
Education [number of case (%)]		0.42	0.08			0.88
9 years	7 (19%)			1 (9%)	1 (13%)	
12 years	12(32%)			4 (36%)	3 (38%)	
>12 years	17 (46%)			6 (55%)	3 (38%)	
Civil status [number of case (%)]		−0.22	0.37			0.38
Live alone	7 (18%)			3 (27%)	4 (50%)	
Live with somebody	30 (79%)			8 (73%)	4 (50%)	
Unknown	1 (3%)					
BMI	23.6 ± 9.5	0.50^*^	0.04	25.8 ± 3.1	22.0 ± 11.8	0.54
Independency (mRS)	1 (0–2)	0.54^*^	0.02	0 (0–1)	1 (0–2)	0.05
Employment [number of case (%)]		−0.01	0.97			0.88
Full-time job	4 (11%)			1 (9%)	2 (25%)	
Part-time job	6 (16%)			3 (27%)	1 (13%)	
Retired/Unemployed	27 (73%)			7 (64%)	5 (63%)	

* *p ≤ 0.05 was considered significant*.

No significant difference with respect to basic characteristics was observed between participants with and without fatigue. The univariate analysis showed that more fatigue significantly correlated to higher BMI and higher independence in the daily activity (mRS) but not to age, gender, civil status, stroke sub-type, education or employment.

### Fatigue

**Figure 1:**
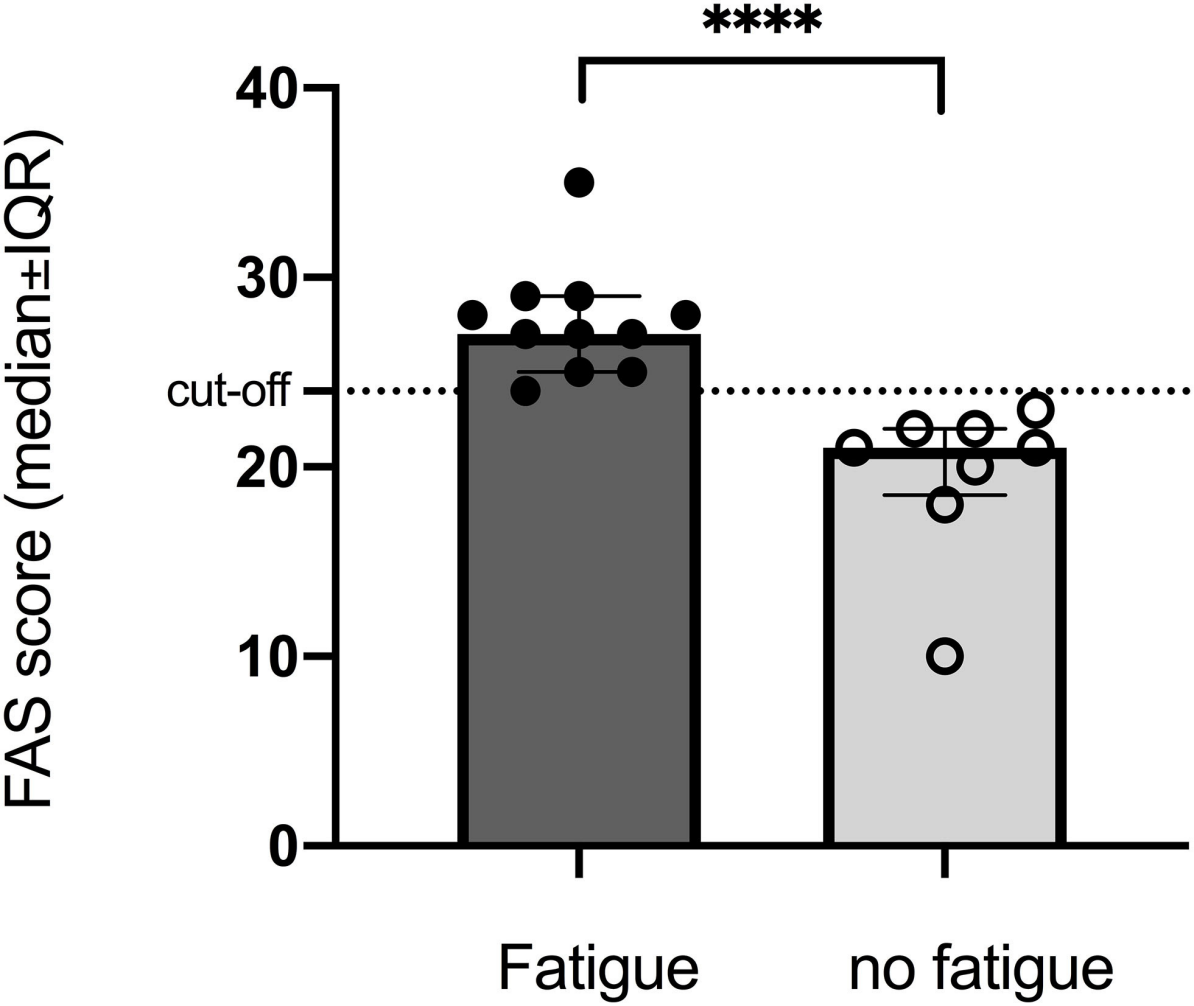
Significant difference between groups with and without fatigue assessed by FAS (cut-off > 24) at 10-years follow-up after stroke onset. ****p < 0.0001.

More than half of the participants suffered from fatigue (FAS > 24) with median score 25 with 25–75% percentile (20–27) even though only half of the participates answered the FAS questionnaire. In the fatigue group, the median score was 27 (24–28). In the non-fatigue group, the median score was 21 (24–28) that was close to the cut-off but significantly lower (*p* < 0.0001) than that in the group with fatigue (Figure 1).

#### Improved Cognition

A weak but significant improvement in global cognition assessed by MMSE (from 27 to 29, *p* = 0.02) was demonstrated among participants at 10-years follow-up compared to the results at 7 months after stroke onset (Table 3). As demonstrated previously (11), visuospatial function (Block design) showed a significant improvement already at 7 months after stroke onset (*p* = 0.0002). This improvement remained after 10 years. Significant improvements on working memory were demonstrated at 10-years follow-up in working memory (Digit span total score) compared to results at 1-week and 7-months post-stroke (*P* < 0.0001). There were no significant differences on process speed and executive function between the follow-up time-points.

**Table 3:** Descriptive results of functional domains, their correlations with fatigue, and comparation between groups with and without fatigue.

**Function domains**					**Univariate correlation** **with fatigue**		**Comparisons between groups** **with and without fatigue**				
**Function domain**	**Assessments**	**Time after stroke onset**	**Total Score**	**Nr**	***r***	***p*-value**	**FAS < 24**	**Nr**	**FAS > 24**	**Nr**	***p*-value**
Global cognition	MMSE	7 months	27 (25-29)	17	0.46	0.14	24 (21-27)	5	28 (26-29)	7	0.08
		10 years	29 (27-30)	38	0.51	0.03^*^	26 (24-29)	8	30 (27-30)	11	0.03^*^
Visuospatial function	WAIS–block design	1 week	27(15-35)	27	0.50	0.10	18 (10-28)	5	36 (6-41)	7	0.43
		7 months	39 (34–51)	19	0.60	0.03^*^	29 (20-35)	5	49 (38–56)	9	0.01^*^
		10 years	36 (29-48)	38	0.48	0.04^*^	32 (20-34)	8	48 (24-50)	11	0.16
Working memory	Digit span for– & backward	1 week	13 (11-14)	29	0.27	0.37	11 (8-14)	6	12 (7-14)	7	0.92
		7 months	13 (12-18)	26	−0.04	0.89	13 (10-17)	7	13 (10-18)	10	0.87
		10 years	17 (14-19)	38	0.51	0.03^*^	16 (12-17)	8	17 (14-19)	11	0.21
Processing speed	TMT-A	1 week	46 (35–56)	27	−0.41	0.21	45 (25-55)	6	41 (26-47)	9	0.67
		7 months	40 (26-65)	11	−0.27	0.55	65 (26-86)	3	42 (40–43)	2	0.80
		10 years	37 (30-50)	37	−0.22	0.37	32 (22-37)	8	37 (30-52)	10	0.11
Executive function	TMT-B	1 week	101 (67–134)	27	−0.48	0.14	102 (66–144)	6	80 (59–257)	9	0.84
		7 months	72 (54–126)	8	0.05	0.92	54 (49–59)	2	135	1	-
		10 years	81 (62–116)	37	−0.30	0.22	60 (47–73)	8	70 (57–141)	10	0.15
Depression	BDI-II	10 years	9 (4-14)	37	0.39	0.10	7 (3-16)	8	11 (4-15)	11	0.25
Anxiety	BAI	10 years	6 (2-11)	35	0.48	0.04^*^	5 (2-7)	8	10 (6-19)	10	0.08

*Score presented as median (25–75% percentile)*.

* *p < 0.05 was considered as significance*.

Univariate correlation analysis revealed that fatigue was significantly associated with better global cognition (MMSE), better working memory and more anxiety at 10-years follow-up after stroke onset as well as better visuospatial function at all three follow-up time points. Fatigue showed negative but not significant correlations with process speed and executive function. Significant differences between groups with and without fatigue, were only observed in global cognition at 10-years follow-up and visuospatial function at 7-months follow-up after stroke onset (Table 3).

### Depression and Anxiety

Twelve participants (*n* =12) were depressed (BDI-II > 13) with median nine with 25–75% percentile (4–14). Approximately one third (*n* = 13) had anxiety problems (BAI > 8) with relatively low median score six with 25–75% percentile (2–11). There was no significant difference on depression and anxiety between groups with and without fatigue (Table 3). Univariate correlation analysis demonstrated that more fatigue was significantly associated with anxiety.

### Linear Regression of Fatigue

A multiple linear regression was calculated to predict fatigue based on BMI, mRS, anxiety (BAI score), MMSE, visuospatial function (Block design) and working memory (Digit span) at 10-years follow-up. Those factors showed significance in the univariate correlation analysis. A significant regression equation was found (*F*_(1, 4)_23.07, *p* = 0.009), with adjusted R^2^ of 0.815, i.e., Block design score at 7-months post-stroke explained 81.5% of fatigue measured with FAS in this model. Participants' fatigue after 10 years was significantly predicted (*p*= 0.009) with an 0.376 increase for each score of Block design at 7-months post-stroke (Table 4).

**Table 4:** Multiple linear regression of fatigue.

**Model**^*******a*******^		**Unstandardized coefficients**		**Standardized** **coefficients**	***t***	**Sig**.	**Correlations**			**Collinearity** **statistics**				
		**B**	**Std. Error**	**Beta**			**Zero-order**	**Partial**	**Part**	**Tolerance**	**VIF**	**Total *R*^**2**^**	**Adjusted *R*^**2**^**	**Durbin-Watson**
1	(Constant)	7,027	3,579		1,964	0,121						0,852	0,815	1,540
	Block design at 7-month	0,376	0,078	0,923	4,803	0,009	0,923	0,923	0,923	1,000	1,000			

a *Dependent Variable: Fatigue*.

**Figure 2:**
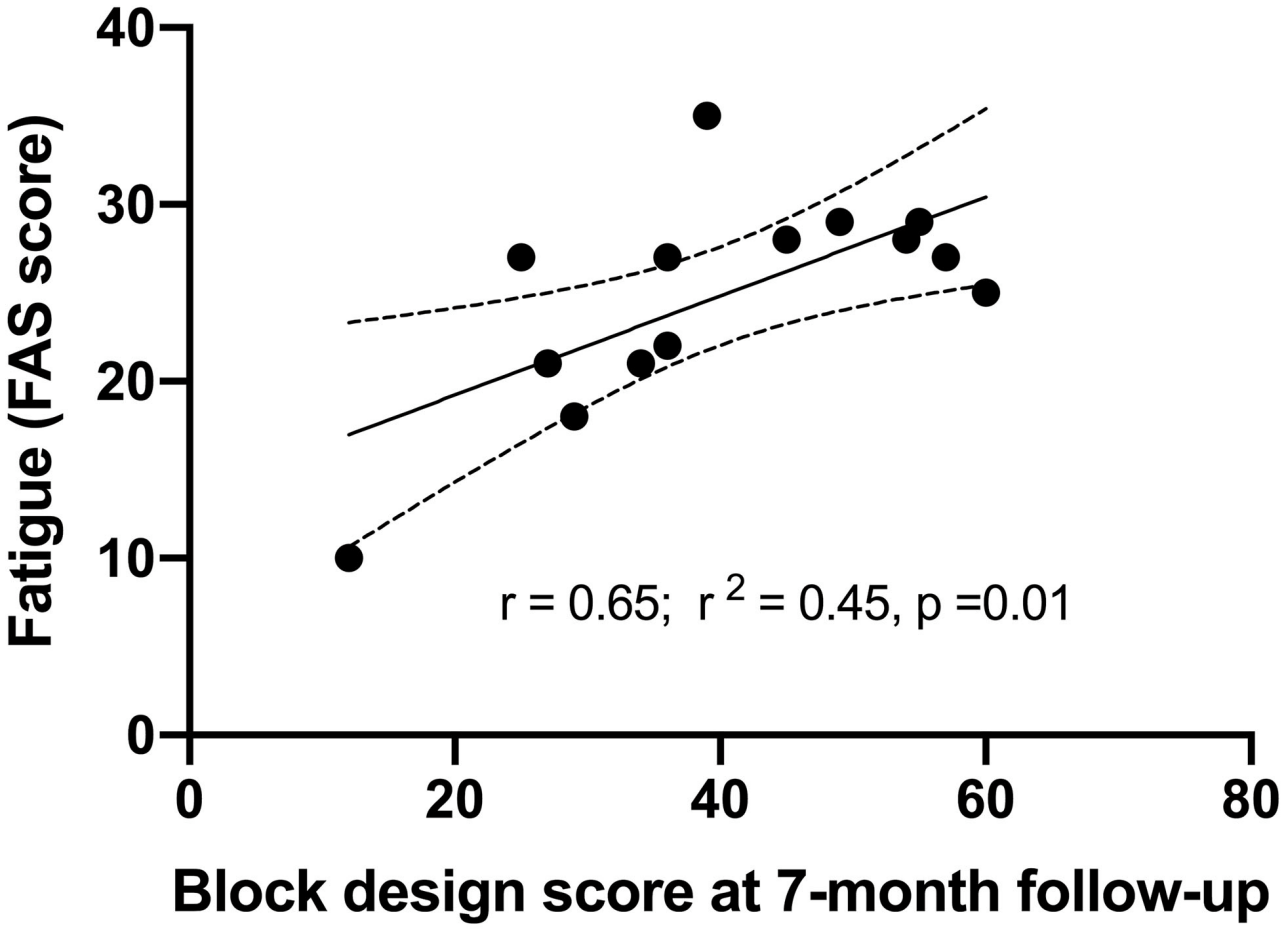
Correlations between fatigue at 10-years follow-up and visuospatial function assessed by Block design at 7-months post-stroke by simple linear regression.

A visual correlation between fatigue and the score of Block design at 7-months follow-up is presented in Figure 2 by simple linear regression analysis (Figure 2). The participants with better performance on Block design had more fatigue assessed by FAS.

## Discussion

This longitudinal study demonstrated more than half of 38 participants suffered fatigue even though most of them were independent in their everyday life 10 years after stroke onset. A significant difference between patients with or without fatigue was observed in visuospatial function at 7-months follow-up and in global cognition at the 10-years follow-up i.e., patients with fatigue performed better. In univariate correlation analyses, higher fatigue score was correlated to higher independency in the daily activity (mRS score), anxiety, higher BMI, better global cognition and better working memory at 10-years follow-up as well as better visuospatial function after 7 months and 10-years. In a multiple regression analysis only visuospatial function at 7-months follow-up was a significant predictor of fatigue 10 years after stroke onset.

More than half of the participants in the current study suffered fatigue at 10-years follow-up. Meanwhile, those who were not considered having fatigue according to FAS also had scores closed to the cut-off. Our results clearly extend the time course of post-stroke fatigue up to 10 years since most of previous studies on post-stroke fatigue shown similar findings up to 2years after stroke onset (6, 29). Because very long-term follow-up has been rarely reported (8), the current finding implies that post-stroke fatigue among stroke survivors may be a very long-term functional impairment possibly with significant negative impact (3–6).

Most of the participants with fatigue performed better in cognitive assessments and daily activity in the present study. This is in line with our previous findings where participants who performed well in the neuropsychological assessment suffered more fatigue (11). The results suggested that these participants may use more energy to be able to achieve better functional outcomes and more independent daily life, but with fatigue as a drawback. Intriguingly, this drawback seems not affect their working capacity since no correlation demonstrated between fatigue and degree of employment in the cohort. Our data imply a dissociation between subjective fatigue and objective fatigability among stroke survivors. This dissociation has previously only been described among other neurological diseases (20, 21). This dissociation may spread light over and explain why the empirical fatigue treatment with balance between activity and rest has been considered as useful clinical recommendation. However, the clinical implication of such dissociation between fatigue and fatigability suggested by Kluger et al. (10) needs further investigation among stroke survivors.

In the current study, no association was observed between fatigue and age, gender, type of stroke, location of stroke, risk factors, education level, civil status, employment, process speed or executive function. This may indicate that post-stroke fatigue has no significant impact on these factors, including social life and return to work, or *vice versa*. On the contrary, anxiety but not depression, showed significant correlation with fatigue in the univariate analysis. There were no significant differences on anxiety between groups with and without fatigue though. This somehow differs from an earlier study where depression was associated with fatigue (22). The discrepancy between these studies may be explained by the variations of the study cohorts.

The unique finding in the current study was that visuospatial function (assessed by Block design) at two latter time-points significantly predicted post-stroke fatigue after 10 years in the correlation analysis. Our results suggest that Block design may be a potential objective assessment to identify and quantify post-stroke fatigue, which requires further study. The association between visuospatial function and fatigue has only been reported in other neurological disorders previously, such as Parkinson and Multiple sclerosis (23, 24), although it remains largely unknown why visuospatial function may predict fatigue. It has been suggested that visuospatial dysfunction and fatigue share some common neural circuits or pathophysiological mechanism (25–28). The common neural circuits may not only present in the right hemisphere stroke but also in the left hemisphere stroke since we recruited equal number of patients with either left- or right hemisphere stroke in the current study. It is also possible the more challenging visuospatial performance (e.g., Block design) may be more sensitive to fatigue than other cognitive domains or tests. This is perhaps because that visuospatial processing in Block design is a fundamental and complex part of human cognition demanding attention and energy (30).

The variable of Block design at 7-months post-stroke was the only parameter that survived multivariate regression analysis, although the univariate analysis reported several parameters associated with fatigue. Our results demonstrated that visuospatial function at 7-months (sub-acute phase), as an independent predictor, could predict large part of fatigue over time at 10-year (chronic phase) post-stroke. A notable long-time gap between predictor and fatigue may offer abroad time window for delivering proper rehabilitation and information about fatigue to the stroke survivors. If this finding could be confirmed by a large cohort study, assessment of Block design at early stage could be used as an independent fatigue predictor. This may enable clinicians to provide important information against fatigue in good time to their patients and patients' relatives for diminishing the negative consequences of post-stroke fatigue.

The strength of the current study was the very long-term follow-up duration with repeated domain-specific cognitive outcomes assessments at acute, sub-acute, and chronic phases over 10 years after stroke onset. This provided us with a distinctive opportunity to study predict/ prognose factors on various outcome over time. However, we are aware of the small number of participants and some variations of the number of participants at different time-points and groups. Together with a single-center study, this makes it hard to generalize the findings to the entire young stroke population. Nevertheless, the current study suggests the possibility of using tests of visuospatial function (e.g., Block design) at sub-acute stage to objectively identify and predict fatigue at chronic stage.

In conclusion, our results extended the time course of post-stroke fatigue up to 10 years after stroke onset. The participants with more fatigue performed better in cognitive assessments and daily activity, which indicated dissociation between fatigue and fatigability among stroke patients. Visuospatial function at the sub-acute phase may be used as a potential objective assessment and an independent predictor of late post-stroke fatigue. This may offer a broad time window for rehabilitation and information about fatigue. The clinical implications of the current findings are worth to be studied further.

## Data Availability Statement

All datasets generated for this study are included in the article/supplementary material.

## Ethics Statement

The studies involving human participants were reviewed and approved by Ethical approval was obtained from the Regional Ethical Review Board in Umeå, Sweden, D-nr 2015/144-31. The patients/participants provided their written informed consent to participate in this study.

## Author Contributions

EE contributed to study conception, sample collection, interpretation of data, and revising the manuscript, provided final approval of the version to be published, and agreed to be accountable for all aspects of the work. XH contributed to study conception, supervision of the acquisition, interpretation of data, drafting and revised the manuscript including the figures, tables, and references, provided final approval of the version to be published, and agreed to be accountable for all aspects of the work.

## Conflict of Interest

The authors declare that the research was conducted in the absence of any commercial or financial relationships that could be construed as a potential conflict of interest.

## Abbreviations

WAIS-IV: Wechsler Adult Intelligence Scale-IV
D-KEFS: Delis-Kaplan Executive Function System
MMSE: Mini mental state examination
mRS: Modified Rankin Scale. BDI-II, Beck Depression Inventory-II
BAI: Beck Anxiety Inventory
FAS: Fatigue assessment scale.
